# The Prefrontal Dectin-1/AMPA Receptor Signaling Pathway Mediates The Robust and Prolonged Antidepressant Effect of Proteo-β-Glucan from Maitake

**DOI:** 10.1038/srep28395

**Published:** 2016-06-22

**Authors:** Hongkun Bao, Pengzhan Ran, Ming Zhu, Lijuan Sun, Bai Li, Yangyang Hou, Jun Nie, Liping Shan, Hongliang Li, Shangyong Zheng, Xiufeng Xu, Chunjie Xiao, Jing Du

**Affiliations:** 1 State Key Laboratory for Conservation and Utilization of Bio-resources in Yunnan, Yunnan University, Kunming, Yunnan, P. R. China; 2 School of Medicine, Yunnan University, Kunming, Yunnan, P. R. China; 3 Beijing Gragen Biotechnology Co. Ltd., Beijing, P. R. China; 4 Department of Psychiatry, The First Affiliated Hospital of Kunming Medical University, Kunming, Yunnan, P. R. China

## Abstract

Proteo-β-glucan from Maitake (PGM) is a strong immune regulator, and its receptor is called Dectin-1. Cumulative evidence suggests that AMPA receptors are important for the treatment of depression. Here, we report that PGM treatment leads to a significant antidepressant effect in the tail suspension test and forced swim test after sixty minutes of treatment in mice. After five consecutive days of PGM treatment, this antidepressant effect remained. PGM treatment did not show a hyperactive effect in the open field test. PGM significantly enhanced the expression of its receptor Dectin-1, as well as p-GluA1(S845) and GluA1, but not GluA2 or GluA3 in the prefrontal cortex (PFC) after five days of treatment. The Dectin-1 inhibitor Laminarin was able to block the antidepressant effect of PGM. At the synapses of PFC, PGM treatment significantly up-regulated the p-GluA1(S845), GluA1, GluA2, and GluA3 levels. Moreover, PGM’s antidepressant effects and the increase of p-GluA1(S845)/GluA1 lasted for 3 days after stopping treatment. The AMPA-specific antagonist GYKI 52466 was able to block the antidepressant effect of PGM. This study identified PGM as a novel antidepressant with clinical potential and a new antidepressant mechanism for regulating prefrontal Dectin-1/AMPA receptor signalling.

Major depression affects millions of people worldwide, it is important to develop a novel and prolonged antidepressant^1^. Proteo-β-glucan from Maitake (PGM), with its unique and complex structure, containing either β-1,6-linked glucan with β-1,3 branches or β-1,3-linked glucan branched with β-1,6 glucosides as a main polysaccharide backbone where a few uncharacterized protein units are attached to it, is the bioactive component of Maitake mushroom (Grifola frondosa)^2^. The medicinal properties of PGM include various physiological benefits ranging from immuno-modulatory and antitumor activities to the treatment of hypertension, diabetes, hypercholesterolemia, viral infections and obesity^2^,^3^. The specific receptor for PGM is dendritic cell-associated C-type lectin-1 (Dectin-1), which is expressed in microglial cells, dendritic cells, monocytes, macrophages, and neutrophils^4^,^5^. Dectin-1 was discovered as the first non-Toll-like receptor. It has been shown that Dectin-1 induces the expression of the anti-inflammatory cytokines IL-2 and IL-10 and stops the inflammation process via Syk-independent pathways^6^. Cumulative evidence has shown that depression is related to immune regulation^7^,^8^. Particularly, the impairment of the normal function of the microglia can lead to depression and impairment of associated neuroplasticity and neurogenesis^9^. However, whether PGM or/and its receptor Dectin-1 are involved in this antidepressant effect remains unknown.

The most famous rapid antidepressant, ketamine, a non-competitive N-methyl-D-aspartate (NMDA) receptor antagonist, was found to rely on increasing α-amino-3-hydroxy-5-methyl-4-isoxazole-propionic acid (AMPA) signalling to exert its antidepressant efficacy^1^,^10^. The AMPA receptor has emerged as a central mediator for the pathophysiology and treatment of depression^11^,^12^,^13^. AMPA receptors, which are tetramers assembled from four subunits, GluA1, GluA2, GluA3 and GluA4, play a key role in the activity-dependent regulation of synaptic strength^11^,^14^,^15^. The mRNA levels of GluA1 and GluA3 in the patients with depression were significantly decreased in the perirhinal cortex and hippocampus^16^. The AMPA receptor potentiator (LY392098) had an antidepressant effect in animal models^17^. The phosphorylation of GluA1 at serine 845 [p-GluA1(S845)] was increased following treatment in mice using the antidepressants flouxetine and tianeptine^18^,^19^, which suggested that phosphorylation of GluA1 might be associated with the potentiation of AMPA receptor signalling^20^. Currently, increasing evidence suggests that AMPA receptors serve as central mediators during the development of the pathophysiology and treatment of depression^16^. However, whether PGM regulates AMPA synaptic plasticity is still unknown.

In this context, we designed a series of behavioural and biochemical experiments to investigate the antidepressant effects of PGM in the animal models of depression. We first studied the effects of various concentrations of PGM on animal models of depression. The expression of Dectin-1 and the role of Dectin-1 in the antidepressant effect were determined after PGM treatment. The phosphorylation of AMPA GluA1 S845 and the expression of AMPA GluA1, GluA2, and GluA3 were also studied in the total protein extract and in the synaptic fraction of the prefrontal cortex (PFC) after PGM treatment. The prolonged effects of PGM after ceasing treatment were also investigated. Moreover, the role of the enhanced AMPA function in the antidepressant effect of PGM was addressed by treatment with an AMPA-specific antagonist, GYKI 52466.

## Results

### PGM demonstrated a robust antidepressant effect in the tail suspension test (TST) and forced swim test (FST)

**Figure 1:**
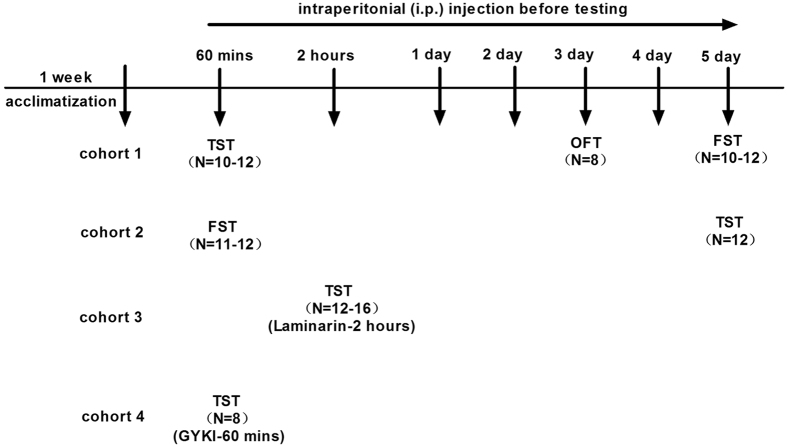
Experimental procedures. Mice were acclimatized for at least 1 week. Four independent cohorts of animals were used to test the antidepressant effects of proteo-β-glucan from Maitake (PGM). The animal behavioural tests were performed 60 minutes (mins) after drugs or vehicle were administered. The first independent cohort of animals underwent the tail suspension test (TST) after 60 minutes treatment, open field test (OFT) on the third day, and forced swim test (FST) on the fifth day. To confirm the antidepressant effects of PGM, the second independent cohort of animals was subjected to the FST after 60 minutes of treatment and to the TST on the fifth day under similar conditions. The third independent cohort of animals was used to test the Dectin-1 receptor-specific blocking reagent Laminarin (Lam) in the antidepressant effect of PGM in the TST. The fourth independent cohort of animals was used to test whether the α-amino-3-hydroxy-5-methyl-4-isoxazole-propionic acid (AMPA) receptor-specific antagonist GYKI 52466 (GYKI) was involved in the antidepressant effect of PGM in the TST.

**Figure 2:**
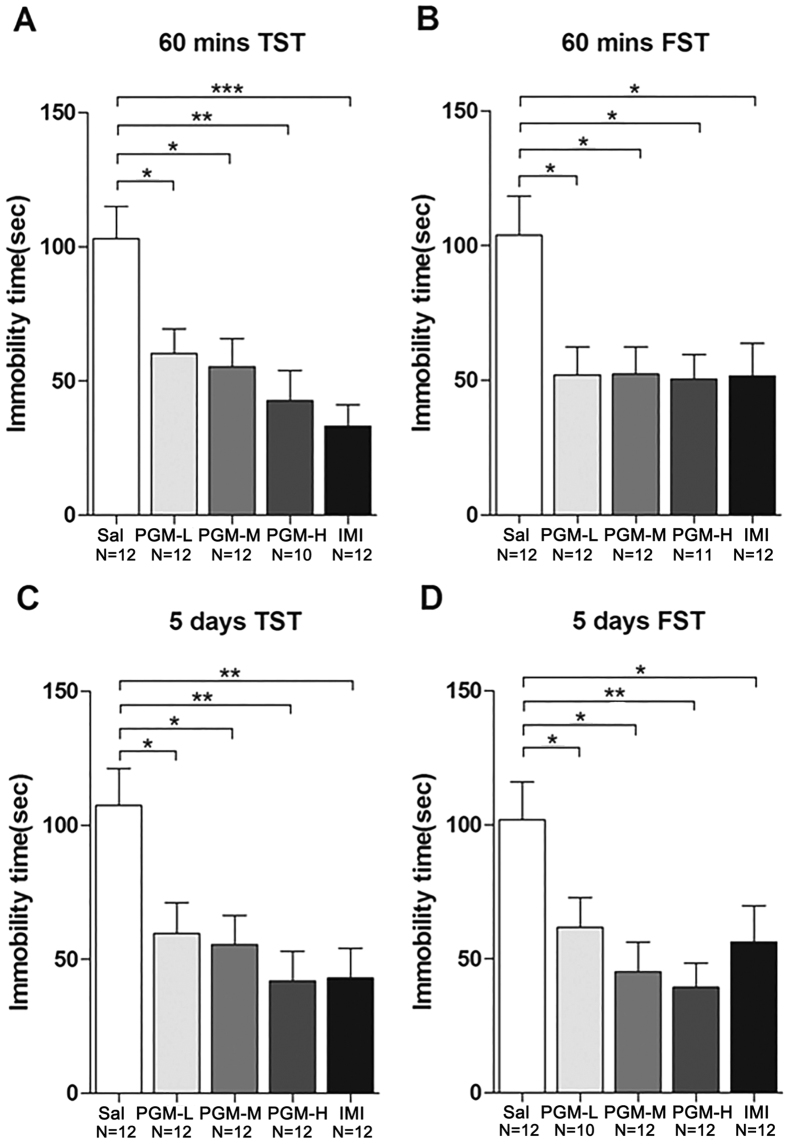
Proteo-β-glucan from Maitake (PGM) demonstrated a significant antidepressant effects in the tail suspension test (TST) and forced swim test (FST). CD-1 mice were i.p. injected with a low dose of PGM (5 mg/kg/day, PGM-L), a medium dose of PGM (8 mg/kg/day, PGM-M), a high dose of PGM (12.5 mg/kg/day, PGM-H), imipramine (15 mg/kg/day, IMI) or saline (Sal). After PGM treatment for sixty minutes (mins) or five days, mice were subjected to the TST or FST. The number (N) of mice per group is indicated in each individual graph. Any experimental data value greater than mean plus 2 × standard deviations (SDs) from a group was considered an outlier and was not considered in the analysis. Data were analysed by one-way ANOVA and presented as the mean ± SE (*post hoc* Tukey’s test, *p < 0.05, **p < 0.01, ***p < 0.001). (**A**,**B**) Sixty minutes after the injection, PGM significantly reduced the immobility time in the TST or FST. (**C**,**D**) After 5 consecutive days of injection, PGM significantly reduced the immobility time in the TST or FST.

Seven-week-old CD-1 mice were intraperitonially (i.p.) injected with a low (5 mg/kg), medium (8 mg/kg), and high (12.5 mg/kg) doses of PGM for 60 minutes or 5 days before testing. Sixty minutes after the treatment, the mice were subjected to either TST or FST (Fig. 1). The data showed that the duration of immobility in the PGM-treated groups was significantly lower than that of the controls (103.0 ± 12.0 sec) in a dose-dependent manner, as low as 60.3 ± 9.2 sec (for 5 mg/kg of PGM), 55.3 ± 10.5 sec (for 8 mg/kg of PGM), and 42.8 ± 11.3 sec (for 12.5 mg/kg of PGM) in the TST (ANOVA, F(4,53) = 6.991, p < 0.01) (Fig. 2A). The positive control imipramine (33.2 ± 8.0 sec) also demonstrated an antidepressant effect (Fig. 2A). To confirm these data, FST was performed under similar conditions. After 60 minutes of treatment with PGM, the data showed that the duration of immobility in the PGM-treated groups was significantly lower than that of the controls (103.8 ± 14.5 sec), as low as 51.0 ± 9.5 sec (for 5 mg/kg of PGM), 52.3 ± 10.0 sec (for 8 mg/kg of PGM), and 50.5 ± 9.1 sec (for 12.5 mg/kg of PGM) for FST, similar to the traditional antidepressant imipramine (51.5 ± 12.2 sec) (ANOVA, F(4,54) = 4.348, p < 0.01) (Fig. 2B). To further examine the antidepressant effect of PGM after long-term treatment, we treated mice with the same doses as mentioned above for 5 consecutive days, and TST and FST were performed after the treatment (Fig. 1). The data showed that the immobility times in the PGM-treated groups were significantly lower than those of the controls (107.3 ± 14.0 sec) in a dose-dependent manner, as low as 60.0 ± 11.6 sec (for 5 mg/kg of PGM), 55.5 ± 11.0 sec (for 8 mg/kg of PGM), and 42.0 ± 11.1 sec (for 12.5 mg/kg of PGM) for TST (ANOVA, F(4,55) = 5.143, p < 0.01) (Fig. 2C). Imipramine (43.1 ± 11.2 sec) also demonstrated an antidepressant effect (Fig. 2C). To confirm these data, FST was performed under similar settings. After 5 days of treatment with PGM, the data showed that the immobility time in the PGM-treated groups was significantly lower than that of the controls (109.1 ± 11.7 sec) in a dose-dependent manner, as low as 61.7 ± 11.1 sec (for 5 mg/kg of PGM), 45.3 ± 11.0 sec (for 8 mg/kg of PGM), and 39.3 ± 9.1 sec (for 12.5 mg/kg of PGM) for FST (ANOVA, F(4,53) = 6.043, p < 0.01) (Fig. 2D). Imipramine (56.3 ± 13.5 sec) also showed a similar antidepressant effect (Fig. 2D).

### PGM did not show locomotor hyperactivity in the open field test (OFT)

**Figure 3:**
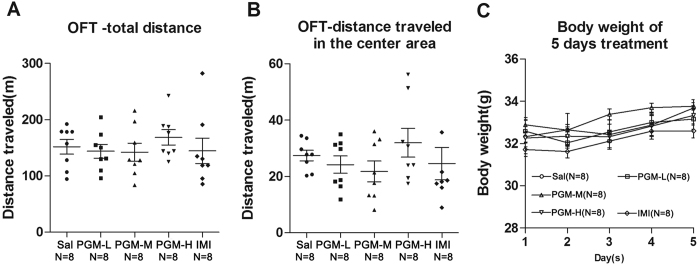
The open field test (OFT) after 3 days of treatment with proteo-β-glucan from Maitake (PGM). CD-1 mice were i.p. injected with a low dose of PGM (5 mg/kg/day, PGM-L), a medium dose of PGM (8 mg/kg/day, PGM-M), a high dose of PGM (12.5 mg/kg/day, PGM-H), imipramine (15 mg/kg/day, IMI) or saline (Sal). After 3 days of treatments, mice were subjected to the OFT. The total distance travelled and the distance travelled in the centre area were determined by the automated tracking system. The number (N) of mice per group is indicated in each individual graph. Data were analysed by one-way ANOVA and presented as the mean ± SE. (**A**) The total distance travelled in the field after PGM treatment. (**B**) The distance travelled in the centre area of the field after PGM treatment. (**C**) Body weight of the 5 consecutive days after PGM treatment.

We performed OFT after 3 consecutive days of treatment with a low, medium, or high doses of PGM (Fig. 1). The total distance travelled showed no significant difference in the PGM-treated groups compared with the controls, which suggested that PGM does not cause locomotor hyperactivity in mice (ANOVA, F(4,35) = 0.4653, p = 0.7607) (Fig. 3A). In addition, the distance travelled in the centre area also showed no significant difference in the PGM-treated groups compared with the control groups (ANOVA, F(4,35) = 0.8818, p = 0.4848) (Fig. 3B). The weights of mice after 5 consecutive days of treatment did not show any significant differences (ANOVA, F(4,35) = 2.448, p = 0.0797) (Fig. 3C).

### PGM mediated the antidepressant effect through its receptor Dectin-1

**Figure 4:**
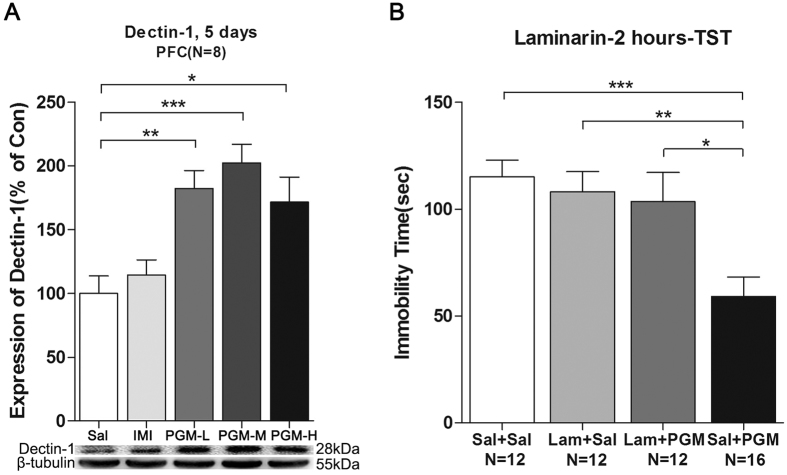
Proteo-β-glucan from Maitake (PGM) mediated the antidepressant effect through its receptor Dectin-1. (**A**) The PGM treatments increased its receptor Dectin-1 levels in the prefrontal cortex (PFC) after 5 days of treatment. CD-1 mice were i.p. injected with a low dose of PGM (5 mg/kg/day, PGM-L), a medium dose of PGM (8 mg/kg/day, PGM-M), a high dose of PGM (12.5 mg/kg/day, PGM-H), imipramine (15 mg/kg/day, IMI) or saline (Sal) for 5 days. Western blot analyses of the proteins from the PFC were performed with anti-Dectin-1 antibodies. (**B**) Dectin-1 receptor specific blocking reagent Laminarin (Lam) was able to block the antidepressant effect of PGM in the tail suspension test (TST). The number (N) of mice per group is indicated in each individual graph. Any experimental data value greater than mean plus 2 × standard deviations (SDs) from a group was considered an outlier and was not considered in the analysis. Data were analysed by one-way ANOVA and presented as the mean ± SE (*post hoc* Tukey’s Test, *p < 0.05, **p < 0.01, ***p < 0.001).

Western blot analyses of total proteins from the PFC were performed with an anti-Dectin-1 antibody, and after 5 days of treatment with PGM, the Dectin-1 levels were significantly increased in the PFC after low (5 mg/kg), medium (8 mg/kg), and high (12.5 mg/kg) doses of the PGM treatment by 182.4 ± 14.1%, 202.4 ± 14.7%, and 171.6 ± 19.6% (ANOVA, F(4,35) = 8.043, p < 0.01) (Fig. 4A), respectively. However, the imipramine-treated group did not show this increase (Fig. 4A). The Dectin-1-specific inhibitor Laminarin (Lam) was used to block Dectin-1 activity (Fig. 1). The pre-treatment with Laminarin almost completely blocked the decrease in immobility time caused by PGM (Sal: 115.2 ± 7.7 sec; Laminarin + Sal: 108.1 ± 9.4 sec; Laminarin+ PGM: 103.5 ± 13.7 sec; PGM: 59.1 ± 9.2 sec) (ANOVA, F(3,48) = 7.479, p < 0.01) (Fig. 4B), which suggested that the Dectin-1 receptor might mediate the antidepressant effect of PGM.

### After 60 minutes of treatment, PGM enhanced AMPA GluA1 S845 phosphorylation in the PFC

**Figure 5:**
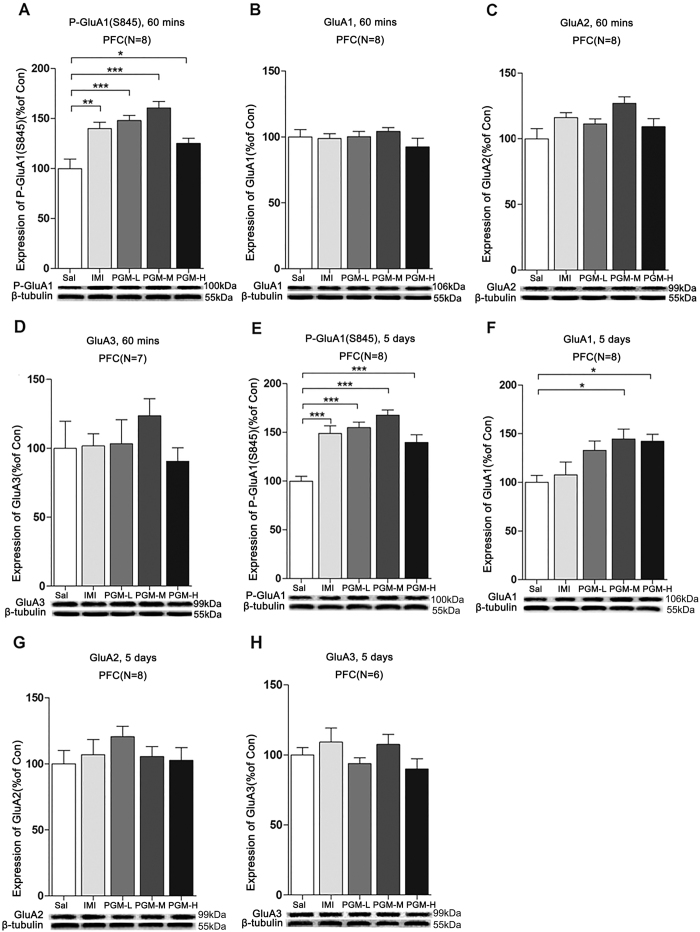
The effects of proteo-β-glucan from Maitake (PGM) on the p-GluA1(S845) (P-GluA1), GluA1, GluA2, and GluA3 levels in the prefrontal cortex (PFC) after 60 minutes and 5 days of treatments. CD-1 mice were i.p. injected with a low dose of PGM (5 mg/kg/day, PGM-L), a medium dose of PGM (8 mg/kg/day, PGM-M), a high dose of PGM (12.5 mg/kg/day, PGM-H), imipramine (15 mg/kg/day, IMI) or saline (Sal) for 60 minutes (mins) or 5 days. Western blot analyses of the proteins from the PFC were performed with anti-p-GluA1(S845), anti-GluA1, anti-GluA2 or anti-GluA3 antibodies. The number (N) of mice per group is indicated in each individual graph. Data were analysed by one-way ANOVA and presented as the mean ± SE (*post hoc* Tukey’s test, *p < 0.05, **p < 0.01, ***p < 0.001). (**A–D**) The expression levels of p-GluA1(S845), GluA1, GluA2 or GluA3 levels in the PFC after 60 minutes of treatment with PGM. (**E–H**) The expression levels of p-GluA1(S845), GluA1, GluA2 or GluA3 in the PFC after 5 days of treatment with PGM.

Previous studies have shown that the phosphorylation of AMPA GluA1 serine 845 represents an important mechanism for the treatment of depression^18^,^19^,^21^. In this context, we further addressed whether the p-GluA1(S845) levels were changed after treatment with PGM. Sixty minutes after i.p. injection, the p-GluA1(S845) levels were significantly increased in the PFC from low (5 mg/kg), medium (8 mg/kg), and high (12.5 mg/kg) doses of PGM by 148.1 ± 5.1%, 160.5 ± 6.5%, and 125.2 ± 5.0%, respectively, similar to that of the imipramine-treated groups (140.1 ± 6.3%) (ANOVA, F(4,35) = 12.14, p < 0.01) (Fig. 5A). However, the levels of total GluA1 (ANOVA, F(4,35) = 0.8392, p = 0.5097), GluA2 (ANOVA, F(3,35) = 2.179, p = 0.0917) or GluA3 (ANOVA, F(4,30) = 0.9194, p = 0.4636) proteins in the PFC remained unchanged (Fig. 5B–D).

### After 5 consecutive days of treatment, PGM increased both the AMPA p-GluA1 (S845) and GluA1 levels in the PFC

After 5 days of treatment with PGM, the level of p-GluA1(S845) was significantly increased in the PFC after low (5 mg/kg), medium (8 mg/kg), and high (12.5 mg/kg) doses of PGM by 155.0 ± 5.6%, 167.6 ± 5.4%, and 139.7 ± 7.8%, respectively, similar to that of the imipramine-treated group (148.8 ± 7.9%) (ANOVA, F(4,35) = 8.611, p < 0.01) (Fig. 5E). It is noteworthy that the PGM treatment also enhanced the levels of total GluA1 by 144.7 ± 10.1% (in the medium-dose group) and 142.2 ± 7.1% (in the high-dose group) (ANOVA, F(4,35) = 8.401, p < 0.01) (Fig. 5F), but not GluA2 (ANOVA, F(4,35) = 1.076, p = 0.3833) or GluA3 (ANOVA, F(4,25) = 1.801, p = 0.1508) expression in the PFC (Fig. 5G,H); however, imipramine had no effect on the total GluA1, GluA2, or GluA3 expression in the PFC (Fig. 5F–H).

### After 60 minutes of treatment, PGM significantly up-regulated AMPA p-GluA1(S845) levels at the synapses in the PFC, similar to imipramine

**Figure 6:**
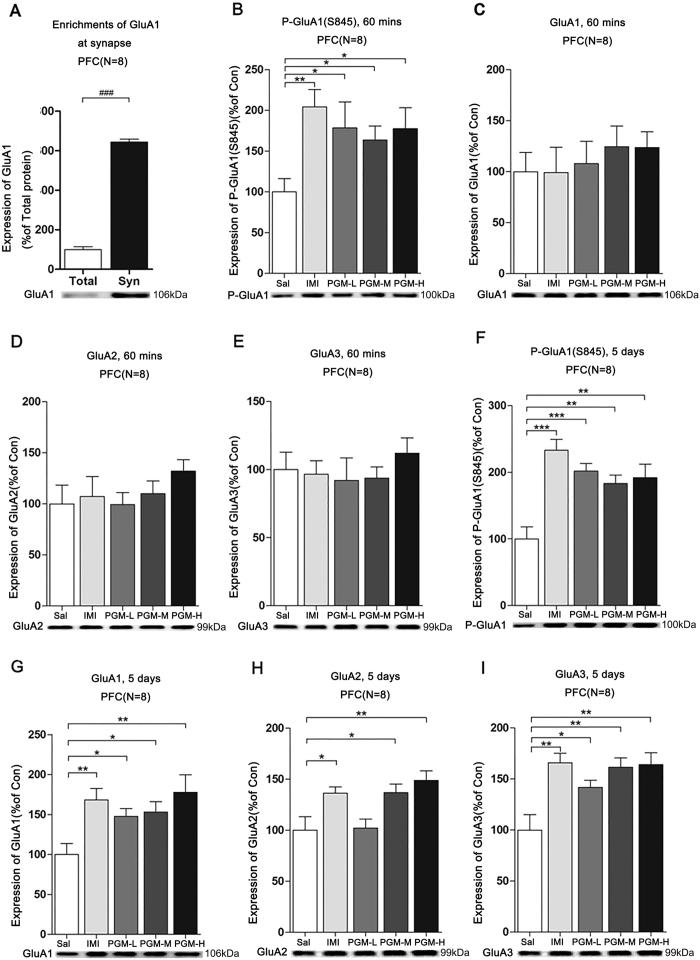
The effects of proteo-β-glucan from Maitake (PGM) on the synaptic p-GluA1(S845) (P-GluA1), GluA1, GluA2 and GluA3 in the prefrontal cortex (PFC). CD-1 mice were i.p. injected with a low dose of PGM (5 mg/kg/day, PGM-L), a medium dose of PGM (8 mg/kg/day, PGM-M), a high dose of PGM (12.5 mg/kg/day, PGM-H), imipramine (15 mg/kg/day, IMI) or saline (Sal) for 60 minutes (mins) or 5 days. Synaptosomal fractions from the PFC were prepared and subjected to western blot analyses with anti-p-GluA1(S845), anti-GluA1, anti-GluA2 or anti-GluA3 antibodies. The number (N) of mice per group is indicated in each individual graph. Data were analysed by two-tailed t-test or one-way ANOVA and presented as the mean ± SE (two-tailed t-test, ^###^p < 0.001; *post hoc* Tukey’s test, *p < 0.05, **p < 0.01, ***p < 0.001). (**A**) Enrichments of GluA1 at synaptosomal (Syn) fractions compared with the control. (**B–E**) The expression levels of synaptic p-GluA1(S845), GluA1, GluA2, GluA3 in the PFC after 60-minute treatment with PGM. (**F–I**) After 5 days of treatment, PGM enhanced synaptic p-GluA1(S845), GluA1, GluA2 and GluA3 levels in the PFC.

To further determine whether the p-GluA1(S845), GluA1, GluA2 and GluA3 levels at the synapses in the PFC were increased, we isolated the synaptic fractions from the PFC. Western blot analysis of p-GluA1(S845), GluA1, GluA2, and GluA3 were performed with the synaptic fractions. We found that the levels of GluA1 were significantly enriched at the synapses compared to the control (Fig. 6A). After 60 minutes of treatment with low (5 mg/kg), medium (8 mg/kg) and high (12.5 mg/kg) doses of PGM, the p-GluA1(S845) levels were increased by 178.4 ± 31.9%, 163.5 ± 17.2%, and 177.6 ± 25.4%, respectively, similar to the imipramine-treated groups (204.3 ± 21.1%) (ANOVA, F(4,35) = 3.044, p < 0.05) (Fig. 6B). However, the levels of GluA1 (ANOVA, F(4,35) = 0.3635, p = 0.8329), GluA2 (ANOVA, F(4,35) = 0.4904, p = 0.7427) and GluA3 (ANOVA, F(4,35) = 1.011, p = 0.4152) proteins at the synapses in the PFC still remained unchanged (Fig. 6C–E).

### After 5 days of treatment, PGM significantly increased p-GluA1(S845), GluA1, GluA2, and GluA3 levels at the synapses in the PFC, similar to imipramine

After 5 days of treatment with PGM, low (5 mg/kg), medium (8 mg/kg), and high (12.5 mg/kg) doses increased the p-GluA1(S845) levels by 201.9 ± 11.5%, 183.2 ± 12.7%, and 192.1 ± 20.2%, respectively, in the PFC (ANOVA, F(4,35) = 9.456, p < 0.01) (Fig. 6F). In addition, in the synaptic fraction from the PFC, the GluA1 levels were increased in a dose-dependent manner by 147.7 ± 9.8% (in the low-dose groups), 153.1 ± 12.8% (in the medium-dose groups), and 177.8 ± 21.8% (in the high-dose groups) (ANOVA, F(4,35) = 4.076, p < 0.01); GluA2 levels were increased by 136.8 ± 8.5% (in the medium-dose groups) and 148.8 ± 9.3% (in the high-dose groups) (ANOVA, F(4,35) = 5.518, p < 0.01); GluA3 levels were increased by 141.8 ± 6.9% (in the low-dose groups), 161.5 ± 9.1% (in the medium-dose groups), and 164.2 ± 11.4% (in the high-dose groups) (ANOVA, F(4,35) = 6.702, p < 0.01), which was similar to imipramine (Fig. 6G–I).

### The antidepressant effect and increased p-GluA1(S845) and GluA1 levels from PGM lasted at least 3 days after stopping treatment with PGM

**Figure 7:**
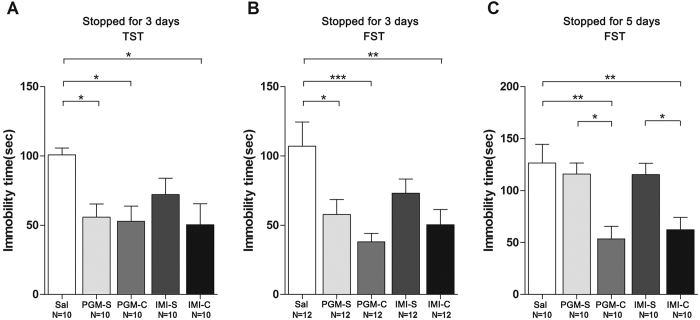
Proteo-β-glucan from Maitake (PGM) produced a prolonged antidepressant effect. CD-1 mice were i.p. injected with a high dose of PGM (12.5 mg/kg/day) or imipramine (15 mg/kg/day, IMI) or saline (Sal) for 5 days. Then, the treatment with PGM or imipramine was stopped for 3 days or 5 days. As shown in the figure, the PGM-S and IMI-S indicated the groups that stopped treatment with PGM or imipramine; PGM-C and IMI-C indicated the groups that continued the treatment. The number (N) of mice per group was indicated in each individual graph. Any experimental data value greater than mean plus 2 × standard deviations (SDs) from a group was considered an outlier and was not considered in the analysis. Data were analysed by one-way ANOVA and presented as the mean ± SE (*post hoc* Tukey’s test, *p < 0.05, **p < 0.01, ***p < 0.001). (**A,B**) After stopping treatment for 3 days, the animals were subjected to TST or FST. (**C**) After stopping treatment for 5 days, mice were subjected to FST.

**Figure 8:**
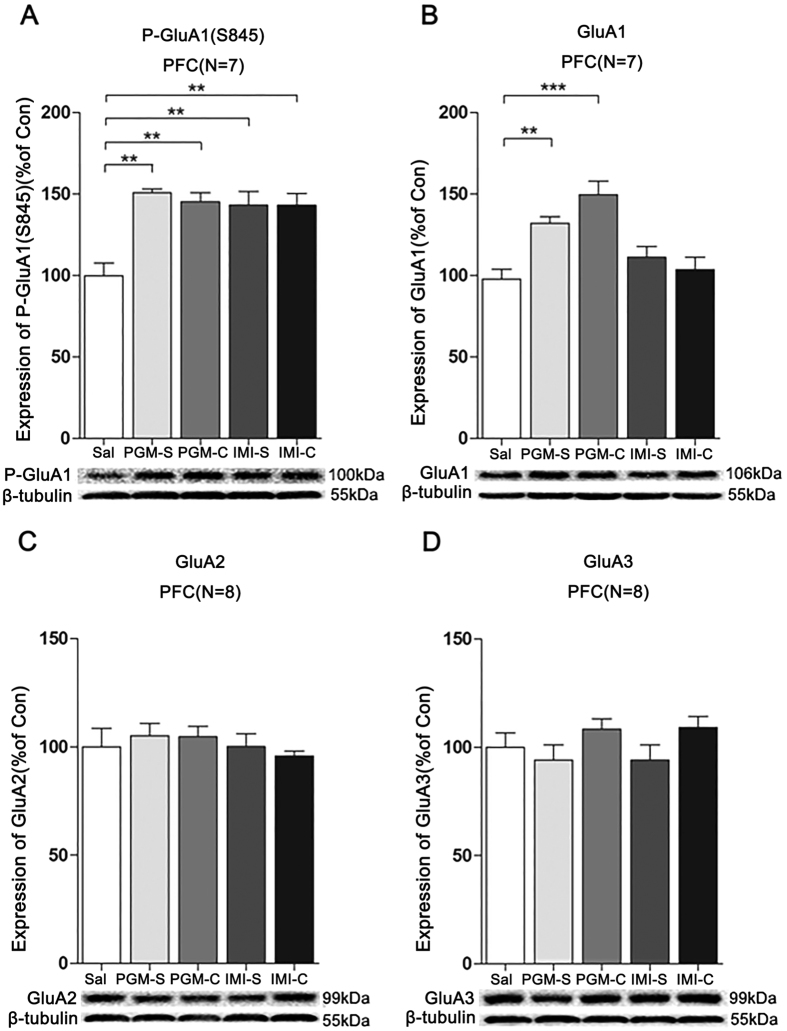
Proteo-β-glucan from Maitake (PGM) evoked a prolonged increase of p-GluA1(S845) (P-GluA1) and GluA1 levels in the prefrontal cortex (PFC). CD-1 mice were i.p. injected with a high dose of PGM (12.5 mg/kg/day) or imipramine (15 mg/kg/day, IMI) or saline (Sal) for 5 days. Then, the treatment with PGM or imipramine was stopped for 3 days. As shown in the figure, the PGM-S and IMI-S indicated the groups that stopped treatment with PGM or imipramine; PGM-C and IMI-C indicated the groups that continued the treatment. Western blot analyses of the proteins from the PFC were performed with anti-p-GluA1(S845), anti-GluA1, anti-GluA2 or anti-GluA3 antibodies. The number (N) of mice per group is indicated in each individual graph. Data were analysed by one-way ANOVA and presented as the mean ± SE (*post hoc* Tukey’s test, *p < 0.05, **p < 0.01, ***p < 0.001). The expression levels of (**A**) p-GluA1(S845), (**B**) GluA1, (**C**) GluA2, or (**D**) GluA3 in the PFC were determined.

Because Burgdorf *et al*. found that ketamine enhanced GluA1 protein expression and Maeng *et al*. found that ketamine showed a long-lasting antidepressant effect^10^,^22^, we hypothesized that the PGM treatment might also result in a prolonged antidepressant effect. After 5 consecutive days of treatment with a high dose of PGM (12.5 mg/kg) or imipramine (15 mg/kg), we stopped treatment for 3 days or 5 days. After stopping treatment for three days, we found that the antidepressant effect of PGM remained in the TST, with 55.8 ± 9.6 sec for the PGM groups compared to the control groups (100.8 ± 5.0 sec) (ANOVA, F(4,45) = 3.627, p < 0.05) (Fig. 7A). The data were confirmed in the FST that the immobility time in the PGM-treated groups with 57.8 ± 10.8 sec after stopping treatment for 3 days was significantly lower than the control group (107.1 ± 17.4 sec) (ANOVA, F(4,55) = 5.204, p < 0.01) (Fig. 7B). After stopping treatment for 3 days, the imipramine group showed an antidepressant effect, but did not reach significance under the same conditions (Fig. 7A,B). Moreover, 3 days after stopping treatment with PGM, the p-GluA1(S845) and GluA1 levels of the PGM groups also remained increased by 150.8 ± 2.4% (ANOVA, F(4,30) = 10.58, p < 0.01) and 132.2 ± 3.9% (ANOVA, F(4,30) = 14.36, p < 0.01) (Fig. 8A,B), respectively, but not GluA2 (ANOVA, F(4,35) = 0.4412, p = 0.7779) and GluA3 (ANOVA, F(4,35) = 1.417, p = 0.2487) (Fig. 8C,D), which suggested that the antidepressant effect was prolonged. However, after 5 days without treatment, the antidepressant effect of PGM was diminished (ANOVA, F(4,45) = 6.841, p < 0.01) (Fig. 7C).

### The antidepressant effect of PGM was blocked by the AMPA receptor-specific antagonist GYKI 52466

**Figure 9:**
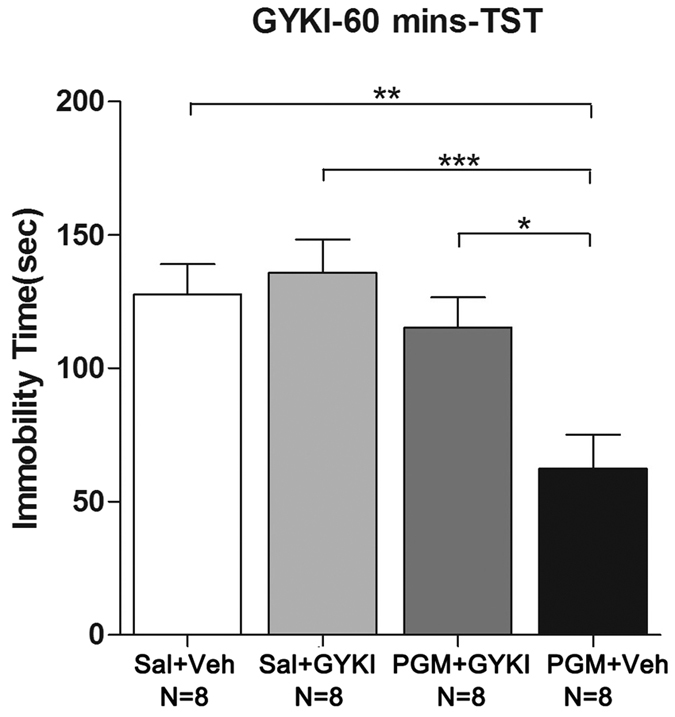
The α-amino-3-hydroxy-5-methyl-4-isoxazole-propionic acid (AMPA) receptor-specific antagonist GYKI 52466 (GYKI) significantly blocked the proteo-β-glucan from Maitake (PGM)-induced an antidepressant effect in the tail suspension test (TST). CD-1 mice were i.p. injected with a medium dose of PGM (8 mg/kg) or saline (Sal) for 60 minutes. GYKI (10 mg/kg) or vehicle (Veh) was administered 30 minutes prior to behavioural testing. Then, the CD-1 mice were subjected to the TST. The immobility time was determined. The number (N) of mice per group is indicated in each individual graph. Data were analysed by one-way ANOVA and presented as the mean ± SE (*post hoc* Tukey’s test, *p < 0.05, **p < 0.01, ***p < 0.001).

We hypothesized that the increase in AMPA receptor signalling is critical for the antidepressant effect of PGM. To test this hypothesis, the AMPA receptor-specific antagonist GYKI 52466 was used to determine whether the antidepressant effect of PGM could be blocked by GYKI 52466. CD-1 mice were i.p. injected with PGM (8 mg/kg) for 60 minutes. GYKI 52466 (10 mg/kg) was administered 30 minutes prior to behavioural testing (Fig. 1). Sixty minutes after the PGM injection, the mice were subjected to TST. The treatment with GYKI 52466 almost completely blocked the decrease of immobility time caused by PGM (Sal: 127.6 ± 11.4 sec; Sal + GYKI: 135.8 ± 12.5 sec; PGM + GYKI: 115.1 ± 11.5 sec; PGM: 62.3 ± 12.8 sec) (ANOVA, F(3,28) = 7.534, p < 0.01) (Fig. 9), which suggested that enhanced AMPA receptor excitability at the synapses might play an important role in the antidepressant effect of PGM.

## Discussion

This study sought to study the robust and prolonged antidepressant effects of PGM and its underlying mechanisms in regulating Dectin-1/AMPA receptor signalling. We found that: (1) PGM had a robust antidepressant effect in the TST and FST after 60 minutes of treatment, and this effect remained after 5 consecutive days of treatment in the TST and FST; (2) PGM had no hyperactive effect in the OFT; (3) PGM enhanced the expression of its receptor Dectin-1 in the PFC after 5 days of treatment; (4) Dectin-1 inhibitor Laminarin was able to block the antidepressant effect of PGM; (5) PGM increased the p-GluA1(S845) levels in the PFC after 60 minutes of treatment and enhanced p-GluA1(S845) and GluA1 expression in the PFC after 5 days of treatment; (6) at the synapses in the PFC, PGM enriched p-GluA1(S845) expression after 60 minutes of treatment and p-GluA1(S845), GluA1, GluA2, and GluA3 levels after 5 days of treatment, similar to imipramine; (7) the antidepressant effect of PGM lasted for 3 days, but not for 5 days after stopping the treatment; and (8) the AMPA receptor-specific antagonist GYKI 52466 was able to block the robust antidepressant effect of PGM.

The Dectin-1/AMPA receptor signalling pathway demonstrated a novel mechanism that is closely related to the neuroimmune system. Currently, the most famous rapid antidepressant is ketamine, a NMDA receptor antagonist that elicits a rapid antidepressant response in patients with depression^23^,^24^ and bipolar depression^25^,^26^,^27^. Evidence suggests that ketamine’s antidepressant properties rely on blocking NMDA receptors, increased AMPA signalling, rapidly induced synaptogenesis^1^,^10^ and lasted for a few days to weeks^28^. Here, we reported a replacement for ketamine, PGM, which showed a robust and prolonged antidepressant effect mediated through the Dectin-1/AMPA receptor signalling pathway. Both PGM and imipramine showed an antidepressant effect in mice (Fig. 2A–D); however, clinical trials are warranted to determine the effective times in humans. Although similar results were found for the 5-day treatment after acute treatment for 60 minutes, a prolonged antidepressant result was observed after stopping the drug treatment for up to three days (Fig. 7A,B). Although PGM showed a prolonged antidepressant effect for 3 days, it was shorter than the weeks-long antidepressant effect of ketamine^10^.

Other psycho-stimulants, such as cocaine and amphetamine, may also have an antidepressant effect, but these psycho-stimulants usually induce excitatory neural toxicity and drug addiction and lead to the development of depressive symptoms during withdrawal^29^,^30^,^31^. Although PGM demonstrated a strong antidepressant effect, it did not elicit hyperactivity like some other psycho-stimulants (Fig. 3A,B). This is different from the psycho-stimulant amphetamine, which significantly increased the total distance travelled in rats during the OFT^32^.

PGM is a proteo-β-glucan. It has been shown that glucan can pass through the blood brain barrier via transporters or during the inflammatory process^5^,^33^,^34^. Here, we reported that PGM within a specific dose range (5–12.5 mg/kg) might bind to its receptor Dectin-1 in the brain to exert a robust antidepressant effect. The fact that the Dectin-1 receptor levels were significantly increased after PGM treatment was an indicator of the activation of the Dectin-1 receptors (Fig. 4A). Dectin-1 inhibition almost completely blocked the antidepressant effect of PGM (Fig. 4B). Therefore, the Dectin-1/AMPA pathway could be a newly discovered pathway for the treatment of depression. Binding to Dectin-1 could lead to the activation of Syk/NFκb signalling pathways and regulation of the neuroimmune system^35^. Targeting Dectin-1 may become a novel strategy for the treatment of mood disorders.

PGM regulated the p-GluA1(S845), GluA1, GluA2, and GluA3 levels at the synapses in the PFC, which suggested a critical role of the AMPA signalling pathway. GluA1 phosphorylation at S845, which is a PKA site, is often viewed as an indicator of GluA1 membrane insertion in neurons and wide channel opening^20^,^36^,^37^,^38^. Previous studies have shown that the levels of AMPA GluA1 in the PFC in depressed patients were decreased^39^,^40^, which was consistent with previous animal experiments^41^,^42^,^43^. AMPA receptors may serve as a common mechanism for the treatment of mood disorders^21^,^44^,^45^,^46^,^47^. Consistent with previous findings, we found that PGM was able to enhance p-GluA1(S845) in the total protein preparations or in the synaptic fractions from the PFC within 60 minutes (Figs 5A and 6B). After 5 days of treatment, PGM was able to enhance both the p-GluA1(S845) and GluA1 levels (Figs 5E,F and 6F,G). It is noteworthy that imipramine had no effect on total GluA1 protein expression, but both PGM and ketamine enhanced GluA1 levels^22^, suggesting a stronger effect in regulating AMPA receptor signalling by PGM (Fig. 5F). Both PGM and imipramine significantly enhanced p-GluA1(S845), GluA1, GluA2, and GluA3 at the synapses in the PFC (Fig. 6F–I). Usually, GluA1/2 and GluA2/3 formed tetramers. Therefore, the three AMPA receptor subunits, GluA1, GluA2, and GluA3, were increased at the synapses after the PGM or imipramine treatment.

As to the mechanisms of regulating AMPA receptors, PGM may go through either direct or indirect mechanism(s). PGM may directly regulate neuronal synapse through the Dectin-1 receptor on the neurons followed by the activation of the signalling cascades (e.g., Syk/NFκb signalling pathway)^35^. PGM may also modulate cytokine expression, leading to the production of IL-2, TNF-α, and IL-18^48^,^49^. It was also reported that cytokines could regulate AMPA receptor signalling^50^. Further exploration of these mechanisms will be one of our future directions.

Previous studies showed that lithium, cordycepin, and dextromethorphanall exerted a rapid antidepressant effect in mice in the TST and FST via up-regulating the AMPA receptor subunits, and an AMPA inhibitor was able to block the antidepressant effects^47^,^51^,^52^. Recent clinical trials have shown that Org 26576 (ionotropic AMPA-type glutamate receptor enhancer) significantly improved symptoms in depressed patients as revealed by the Montgomery-Asberg Depression Rating Scale^53^. In addition, the biaryl-propyl-sulphonamide ARPs (LY392098 and LY451616, AMPA receptor potentiators) have antidepressant effects in animal models of depression, in learned-helplessness models of depression, and in animals exposed to chronic mild stress^17^,^54^, which suggests that the enhancement of AMPA function was sufficient for the antidepressant effect. We found that PGM up-regulated AMPA receptors at the synapse (Fig. 6G–I), and this effect lasted for 3 days after stopping the treatment (Fig. 8B). The AMPA antagonist GYKI 52466 was able to block the PGM-induced antidepressant effect (Fig. 9), which suggested that enhanced AMPA synaptic transmission is essential for the antidepressant effect.

In this paper, we identified PGM as a strong antidepressant with clinical potential for the treatment of depression. It acts by enhancing novel prefrontal Dectin-1/AMPA receptor signalling. This study suggested that the novel target Dectin-1 may be significant in the development of effective antidepressants with novel mechanism(s) for the treatment of major depression.

## Methods

### Animals

All animal procedures were carried out in accordance with the Guide for the Care and Use of Laboratory Animals (ISBN: 0-309-05377-3) and were approved by the Institutional Animal Care and Use Committee at Yunnan University School of Medicine (IACUC: MS201402). Male CD-1 mice (6 weeks; starting weight, 22–26 g; Vital River, Beijing, China) were group housed (N = 4/cage) in an animal room with a constant temperature (22 ± 1 °C) and maintained on a 12-hour light/dark cycle (lights on/off at 9:00 A.M./9:00 P.M.), with constant humidity (55 ± 10%) and free access to water and food. After a one-week acclimatization period, the mice were treated with drugs or vehicle in a volume of 10 μl/g by intraperitoneal (i.p.) injection and tested between 9 A.M. and 12 A.M.

### Bioactive Proteo-β-Glucan from Maitake (PGM)

The Mataike D-fraction was extracted from Maitake mushrooms, which were prepared in a standardized procedure as described previously by Mushroom Wisdom, Inc.^3^,^55^. The D-fraction obtained using this standard method showed a single peak with a MW of 1200–2000 kDa by HPLC analysis and consisted of 98% polysaccharide and 2% peptides. We purchased the supplies from Mushroom Wisdom Inc (East Rutherford, NJ, USA). They labeled their product as containing 30% of the D-fraction solution in water and glycerol. We performed a triple volume of 95% ethanol precipitation procedure to remove the water and glycerol. After incubating the mixture for 60 minutes at room temperature, the samples were centrifuged at 12000rcf/min at 4 °C for 60 minutes^35^. The precipitate was dried with air and re-dissolved in saline for the animal i.p. injections. After ethanol extraction, the resulting proteo-β-glucan was defined as the proteo-β-glucan from Maitake (PGM).

### Animal behavioural studies

To examine the antidepressant effects from PGM, mice were randomly assigned to five treatment groups: saline (sterile 0.9% sodium chloride solution), low dose of PGM (5 mg/kg, dissolved in saline), medium dose of PGM (8 mg/kg, dissolved in saline), high dose of PGM (12.5 mg/kg, in saline), and imipramine (15 mg/kg, in saline; Sigma, St. Louis, MO, USA). The animal behavioural tests were performed 60 minutes after drugs or vehicle were administered, and the TST was performed after 60 minutes, OFT on the third day, and FST on the fifth day. To confirm the antidepressant effects of PGM, another group of mice was subjected to the FST after 60 minutes and to the TST on the fifth day, respectively, under similar conditions.

To examine whether the antidepressant effect of PGM could be blocked by Laminarin (Lam, a specific Dectin-1 blocking reagent, Sigma, St. Louis, MO, USA), CD-1 mice were i.p. injected with Laminarin (10 mg/kg) 2 hours prior to the behavioural testing, followed by the PGM injection one hour later. Sixty minutes after the PGM injection, the mice were subjected to the TST^56^. The AMPA-specific inhibitor GYKI 52466 (a selective non-competitive AMPA receptor antagonist, TOCRIS Bioscience, R&D, Minneapolis, USA) was used to determine the AMPA effect. CD-1 mice were i.p. injected with PGM (60 minutes before the TST) followed by a GYKI 52466 injection (10 mg/kg in 8%DMSO/92%saline 30 minutes before the TST)^47^,^57^. For each drug treatment, the control mice received the vehicle alone via i.p. injection.

### Tail suspension test (TST)

The TST was performed according to a previously described procedure^58^ with minor modifications. A piece of tape 7 cm in length and 2 cm in width was positioned with approximately 2 mm of the tail protruding. Each mouse was individually suspended by the tail from a bar (30 cm high) and videotaped during a 6-minute test session. Immobility time was quantified by a naive observer for the last 4 minutes.

### Open field test (OFT)

An activity chamber (60 × 60 × 30 cm) with a black floor was divided into 16 squares of equal area (15 × 15 cm) by white lines and used to study PGM-induced locomotor hyperactivity. Three days after i.p. injection with various concentrations of drugs, the mice were placed in the centre of the chamber and their behaviours were recorded for 60 minutes. The total distance travelled and the distance travelled in the centre area (the 4-square area in the middle of the chamber) were analysed by the ANY-maze system (Stoelting, Wood Dale, USA).

### Forced swim test (FST)

The FST was carried out according to previously described procedures^59^ with minor modifications. Mice were placed in a cylinder (Φ = 20 cm) with water (temperature between 23 ± 1 °C) 20 cm in depth. Mice were videotaped during a 6-minute test session and were later analysed for mobility for the last 4 minutes. Mobility was defined as any movement beyond what was necessary to maintain their head above water. Immobility time was quantified by a naive observer.

### Western blot analysis

The PFC tissue was lysed with an ice-cold radioimmunoprecipitation assay (RIPA) buffer [20 mM Tris (pH7.5), 150 mM NaCl, 1% Triton X-100, sodium pyrophosphate, β-glycerophosphate, EDTA, Na_3_VO_4_, leupeptin plus a Protease Inhibitor Cocktail Tablet (Roche, Mannheim, Germany), and Phosphatase Inhibitor Cocktail Tablet (Roche, Mannheim, Germany)] in a tissue grinder (Wheaton, Millville, USA). Protein concentrations were determined using a BCA protein assay kit (Pierce Biotechnology, Rockford, USA). Equal amounts of proteins were subjected to 7–10% SDS-PAGE gel electrophoresis and transferred to the polyvinylidene difluoride (PVDF) membranes (Pall, New York, USA). The antibodies against Dectin-1 (Rat mAb, 1:200, Santa Cruz, Dallas, USA), p-GluA1(S845) (Rabbit mAb, 1:1000, Cell Signaling Technology, Boston, USA), GluA1 (Goat pAb, 1:1000, Santa Cruz, Dallas, USA), GluA2 (Rabbit mAb, 1:2000, Abcam, Cambridge, UK), and GluA3 (Rabbit mAb, 1:5000, Abcam, Cambridge, UK) were used. The secondary antibodies were horseradish peroxidase-conjugated goat anti-rat (1:2000, Affinity Bioscience, Cincinnati, USA), goat anti-rabbit (1:5000, Affinity Bioscience, Cincinnati, USA) or donkey anti-goat antibodies (1:5000; Santa Cruz, Dallas, USA). The antibody for β-tubulin (Mouse mAb, 0.2 μg/ml, Affinity Bioscience, Cincinnati, USA) was applied for loading calibration. The immunoreactive bands were visualized using the ECL detection system (Millipore, Billerica, USA). The images were acquired by the FlourChem E image system (FE0511, ProteinSimple, San Jose, USA) and quantified by ImagePro Plus version 6.0 software (Media Cybernetics, Rockville, USA).

### Synaptosomal preparation

The synaptosomal fractions were prepared from the PFC tissue using the differential and discontinuous Ficoll gradient centrifugation method^44^,^45^,^60^. Tissue was homogenized by a tissue grinder and then an electronic polytron homogenizer (AutoScience, Tianjin, China) in cold Syn buffer (300 mM mannitol and 1 mM EDTA, pH 7.4). The crude homogenates were centrifuged at 5000× *g* for 10 minutes, and the supernatants were centrifuged again at 15000× *g* for 30 minutes. The resuspended pellets were loaded on Ficoll gradient (2%, 8%, 12%, 16%, 20%) tubes and centrifuged at 22000× *g* for 90 minutes. The 8/12% and 12/16% interfaces were carefully removed, diluted in Syn buffer at a ratio of 1:4, and centrifuged for 20 minutes at 15000× *g*. The pellets were resuspended and lysed with lysis buffer. All samples were constantly maintained at 4 °C during all steps. Western blot was performed to analyse the synaptic expressions of the p-GluA1(S845), GluA1, GluA2, and GluA3.

### Statistical analysis

All data were analysed by one-way analysis of variance (ANOVA) and *post hoc* Tukey’s tests and presented as the mean ± SE via SPSS version 17. Any experimental data value greater than mean plus 2 × standard deviations (SDs) from a group was considered an outlier and was not considered in the analysis. A p-value less than 0.05 was considered significant. Figures were generated by GraphPad Prism version 5 software.

## Additional Information

**How to cite this article**: Bao, H. *et al*. The Prefrontal Dectin-1/AMPA Receptor Signaling Pathway Mediates The Robust and Prolonged Antidepressant Effect of Proteo-β-Glucan from Maitake. *Sci. Rep.* **6**, 28395; doi: 10.1038/srep28395 (2016).
